# Working Memory Capacity Limits Motor Learning When Implementing Multiple Instructions

**DOI:** 10.3389/fpsyg.2017.01350

**Published:** 2017-08-22

**Authors:** Tim Buszard, Damian Farrow, Simone J. J. M. Verswijveren, Machar Reid, Jacqueline Williams, Remco Polman, Fiona Chun Man Ling, Rich S. W. Masters

**Affiliations:** ^1^Institute of Sport, Exercise and Active Living, Victoria University, Melbourne VIC, Australia; ^2^Game Insight Group, Tennis Australia, Melbourne VIC, Australia; ^3^Skill Acquisition, Australian Institute of Sport, Canberra ACT, Australia; ^4^Institute for Physical Activity and Nutrition (IPAN), School of Exercise and Nutrition Sciences, Deakin University, Geelong VIC, Australia; ^5^School of Exercise and Nutrition Sciences, Queensland University of Technology, Brisbane QLD, Australia; ^6^Faculty of Health, Sport and Human Performance, University of Waikato Hamilton, New Zealand; ^7^Department of Psychology, Bournemouth University Poole, United Kingdom; ^8^School of Public Health, Li Ka Shing Faculty of Medicine, The University of Hong Kong Hong Kong, Hong Kong

**Keywords:** working memory capacity, motor skill acquisition, instructions, explicit learning, children’s motor learning

## Abstract

Although it is generally accepted that certain practice conditions can place large demands on working memory (WM) when performing and learning a motor skill, the influence that WM capacity has on the acquisition of motor skills remains unsubstantiated. This study examined the role of WM capacity in a motor skill practice context that promoted WM involvement through the provision of explicit instructions. A cohort of 90 children aged 8 to 10 years were assessed on measures of WM capacity and attention. Children who scored in the lowest and highest thirds on the WM tasks were allocated to lower WM capacity (*n* = 24) and higher WM capacity (*n* = 24) groups, respectively. The remaining 42 participants did not participate in the motor task. The motor task required children to practice basketball shooting for 240 trials in blocks of 20 shots, with pre- and post-tests occurring before and after the intervention. A retention test was administered 1 week after the post-test. Prior to every practice block, children were provided with five explicit instructions that were specific to the technique of shooting a basketball. Results revealed that the higher WM capacity group displayed consistent improvements from pre- to post-test and through to the retention test, while the opposite effect occurred in the lower WM capacity group. This implies that the explicit instructions had a negative influence on learning by the lower WM capacity children. Results are discussed in relation to strategy selection for dealing with instructions and the role of attention control.

## Introduction

Working memory (WM) is responsible for holding information in a highly active state in mind, often in the face of interference (Baddeley and Hitch, 1974; Miyake and Shah, 1999; Kane et al., 2001). The limited capacity of WM is well documented (Cowan, 2010; Engle, 2010; Logie, 2011), with only a set amount of information or stimuli maintained in an active state at any given time. The importance of WM capacity to human cognition is exemplified by its remarkable predictive power on complex cognitive skills, such as reading comprehension (e.g., Daneman and Carpenter, 1980), problem solving (e.g., Seyler et al., 2003) and general intelligence (e.g., Engle et al., 1999).

Working memory is not restricted to cognitive tasks, however, as practicing and learning motor skills can also demand WM involvement – whether it is the conscious correction of movement errors in an attempt to develop strategies about how to perform a skill (Maxwell et al., 2003), the sequencing of movements such as a dance routine (Cortese and Rossi-Arnaud, 2010), or the implementation of coaching instructions (Liao and Masters, 2001). In each of these scenarios, WM is required to hold the relevant information (i.e., previous errors, the order of a movement sequence, or the instructions) whilst simultaneously performing the skill. Evidence that WM is involved when performing movements can also be derived from studies examining children’s ability to carry out instructions. When multiple instructions were provided, the ability to enact the instructions was positively associated with WM capacity (Engle et al., 1991; Gathercole et al., 2008; Jaroslawska et al., 2016; Waterman et al., 2017). The belief is that environments that place high demands on WM will manifest in superior learning for individuals with a larger WM capacity (for similar arguments, see Steenbergen et al., 2010; Capio et al., 2012; van Abswoude et al., 2015). However, this is yet to be substantiated with regards to motor learning. Accordingly, examining the effect of practice that places high demands on WM was the primary aim of the current study

The results of studies in children and older adults – two populations that typically possess lower WM capacity compared to the average young adult – offer indirect support for the assertion that WM capacity acts as a constraint on motor learning when the practice conditions places high demands on WM. For both groups, motor performance improved significantly more when practice was purported to minimize WM involvement via the reduction of errors during early practice, as opposed to when errors were frequent (Chauvel et al., 2012; Capio et al., 2013a,b; Maxwell et al., 2017). However, these studies focused on motor skill performance/learning without measuring the WM capacity of participants. Hence, the results only offer speculative support for the link between WM capacity and motor learning. Stronger evidence for this relationship was offered by a study examining the learning of a finger-tapping motor sequence (Bo and Seidler, 2009). For this task, adult participants were explicitly aware of the sequence being acquired, which presumably taxed WM resources. Notably, positive associations between WM capacity and the rate of learning were reported, illuminating the benefits of higher WM capacity under conditions demanding WM. However, given that Bo and Seidler (2009) assessed adult participants, it is unclear whether these results can be extrapolated to children. Moreover, Bo and Seidler (2009) examined motor learning in a simple sequencing task as opposed to a gross motor skill in a real-world setting. Hence, further evidence of the relationship between WM capacity and motor learning is required in more ecologically valid environments.

Assessing the demands placed on WM during motor skill practice has typically been assessed via two methods. The most common approach has involved asking participants, following a period of practice, to execute the motor skill while concurrently performing a cognitively demanding secondary task (e.g., Liao and Masters, 2001; Maxwell et al., 2003). The secondary task is thought to demand WM; hence, if motor skill performance declines when performing the secondary task, the learner is assumed to have become reliant on using WM to execute the motor skill. Thus, the preceding practice is thought to have emphasized use of WM when performing the motor skill. Displaying poor ability to execute a motor skill whilst concurrently performing a secondary task is consistently found following engagement in practice that features frequent errors (Maxwell et al., 2001; Poolton et al., 2005; Chauvel et al., 2012; Capio et al., 2013a,b) or the provision of multiple explicit instructions (Liao and Masters, 2001; Poolton et al., 2006a; Masters et al., 2008; Lam et al., 2009). It is therefore assumed that these practice conditions place high demands on WM. However, this approach only provides an indirect assessment of the demands on WM during practice. An alternative method is to measure participants’ reaction time to an external probe (e.g., a loud beep) when performing the motor skill. When WM is engaged in a task, reaction times to an external probe are elongated (Koehn et al., 2008; Lam et al., 2010a,b). This was demonstrated in a basketball task, during which participants’ reaction times were longer during practice that featured frequent errors (Lam et al., 2010b). It was suggested that participants were using their WM to test hypotheses in an attempt to solve performance of the skill. Measuring reaction times to an external probe therefore provides an indication of the demands placed on WM during practice.

In the current study, we aimed to identify whether WM capacity influenced children’s learning of a gross motor skill (basketball shooting) under practice conditions that emphasized WM involvement via the repeated provision of explicit technical instructions about the skill. At the core of our hypotheses was the expectation that children with lower WM capacity would have more difficulty maintaining the instructions in the foci of attention and this would consequently restrict the ability to implement the instructions. We therefore predicted that the children with lower WM capacity would display inferior motor performance to their peers with higher WM capacity following provision of the instructions. Specifically, we hypothesized that children with lower WM capacity, when compared to their peers with higher WM capacity, would display: (a) poorer compliance with the instructions over a period of practice; (b) a reduced ability to verbally recall the instructions when prompted; and (c) smaller improvements in motor performance following practice. Moreover, we expected these differences to become apparent from the beginning of the intervention when the instructions were first provided. This was based on the assertion that the provision of explicit technical instructions would have an immediate positive impact on performance (e.g., Lam et al., 2009). In line with previous studies, we also hypothesized that all children, irrespective of WM capacity, would display poorer performance when required to concurrently perform a cognitive secondary task during post-testing, as all children were expected to become reliant on using the instructions to perform the skill more successfully. Finally, we expected reaction times to an external probe (also referred to as probe reaction times) to be elongated following exposure to the instructions.

## Materials and Methods

### Participants

One-hundred and eleven children (60 boys, 51 girls) from grades three and four in primary school volunteered to participate in the study. Children provided informed assent to participate, whilst parents/guardians provided informed consent. The Human Research Ethics Committee of Victoria University (Melbourne) approved the study. Twenty-one children were excluded from the study because they: (a) had played or were playing organized basketball at the time of the study (*n* = 18), (b) did not speak English (*n* = 1), (c) declined to participate in the working memory assessment (*n* = 1), or was absent from school during testing days (*n* = 1). The mean age of the remaining sample (*n* = 90) was 9 years and 6 months (*SD* = 6 months; minimum = 8 years 0 months; maximum = 10 years 7 months). Only children who fell into the lowest (low WM capacity) and highest (high WM capacity) thirds on the composite score of verbal WM capacity (see Cognitive Assessments) were required to participate in the motor learning task (see **Table 1** for participant details). Extreme group design experiments are commonplace in working memory capacity research (e.g., Kane et al., 2001) and are effective for increasing statistical power.

**Table 1:** Difference between the two experimental groups (mean ± standard deviation)

		Lower WM capacity	Higher WM capacity	*t-value*	*p-value^∗^*
	*N*	24	24	–	–
	Gender breakdown	15 boys, 9 girls	14 boys, 10 girls	–	–
	Age	9.7 ± 0.5	9.3 ± 0.7	2.4	0.02
Verbal WM	Listening Recall	8.8 ± 2.5	14.4 ± 3.2	6.8	<0.001
	Counting Recall	13.0 ± 1.9	23.2 ± 2.4	16.2	<0.001
	Composite Score	–1.0 ± 0.5	1.0 ± 0.5	13.7	<0.001
Visuo-spatial WM	Spatial Recall	13.1 ± 4.1	21.5 ± 5.9	5.7	<0.001
	Odd One Out	16.3 ± 3.9	24.0 ± 4.2	6.6	<0.001
	Composite Score	–0.8 ± 0.6	0.7 ± 0.8	7.3	<0.001
Attention	Score!	7.1 ± 2.1	8.6 ± 1.3	2.9	0.01
	Score!DT	13.0 ± 3.6	15.7 ± 1.9	3.2	0.009

^∗^*p-values adjusted for multiple comparisons using the Holms method with alpha set to 0.05*.

### Cognitive Assessments

All children were assessed on four measures of WM and two measures of attention. Each child was assessed individually in quiet areas of the schools by the same experimenter (SV), with each session lasting approximately 60 min. The WM measures were extracted from the Automated Working Memory Assessment (Alloway, 2007), while the attention measures were taken from the Test of Everyday Attention for Children (Manly et al., 2001). Variables in addition to verbal WM capacity were measured in an attempt to provide a more comprehensive understanding of the cohort of participants.

#### Verbal WM Capacity

The Listening Recall Task and the Counting Recall Task were used to assess verbal WM capacity. For the Listening Recall Task, children were presented with spoken sentences and were required to say whether the sentences were “true” or “false” and then recall the final word of the sentence (e.g., ‘dogs have four legs’; the answer is true and legs). If children responded correctly on sufficient trials (4 out of 6), the number of sentences increased. For the Counting Recall Task, children were presented with sets of shapes and were required to count aloud the number of red circles that appeared on the screen (the number of red circles varied between 4 and 7). Afterward, children had to recall the number of red circles in each set of shapes in the correct sequence (e.g., 6-4-7). Task difficulty increased when children responded correctly on sufficient trials (4 out of 6), and this was achieved by adding one more set of shapes. The raw scores on each task were recorded with possible scores ranging from 0 to 40. From these two tasks, a composite score of verbal WM capacity was calculated. This was achieved by z-transforming the raw scores in each task and then computing the average of the two z scores. Z transformation is a common approach to calculate a composite WM score when multiple WM tasks are used (e.g., Unsworth et al., 2012).

Verbal WM capacity was selected as the variable to divide children into higher and lower WM capacity groups. This was because the verbal system within WM, as opposed to the visuo-spatial system, has been associated with the ability to follow instructions (Jaroslawska et al., 2016). Likewise, positive correlations have also been revealed between verbal WM capacity and neural activity in a region of the brain associated with explicit motor learning (Buszard et al., 2016).

#### Visuo-Spatial WM Capacity

The Spatial Recall Task and the Odd One Out Task were administered as measures of visuo-spatial WM capacity. In the Spatial Recall Task, children viewed two shapes; the shape on the left was always positioned in an upright position; however, the shape on the right was presented in various angles. The children were required to determine whether the shape on the right was the same as or opposite as the shape on the left. Additionally, the shape on the right featured a red dot and the children had to remember the position of the dot (or, when more than one set of shapes appeared, the position of several dots). Children had to immediately respond after the sentence with “same” or “opposite”, and then recall the position of each red dot in the correct sequence after the final shape was presented. Task difficulty was heightened when children responded correctly on sufficient trials (4 out of 6) by increasing the number of shapes that were presented. For the Odd One Out Task, children were presented with a static view of three shapes and were immediately required to indicate which shape was the odd one out. Importantly, children were required to remember the location of each odd shape (i.e., left, middle, right) during each trial and then recall the position of each shape after the final shape was presented. Task difficulty was manipulated by increasing the number of shapes presented, and this occurred when children responded correctly on sufficient trials (4 out of 6). The raw scores were recorded, with the range of possible scores on the two tasks being 0 to 40. A composite score for visuo-spatial WM capacity was calculated in the same manner as verbal WM capacity.

#### Attention

The two measures of attention were Score! and Score!DT. Score! measured the ability to sustain attention on a single stimulus. Children were required to count the number of auditory beeps (345 ms duration), which varied between 9 and 15 beeps across 10 trials. Each beep was separated by an interval that varied between 500 to 5000 ms. The raw score was recorded, with possible scores ranging from 0 to 10. Score!DT was an extension of Score! as it measured the ability to sustain attention on multiple stimuli. The same protocol as Score! was adopted, except children were also asked to listen to a news report that was played concurrently with the beeps. Children were specifically instructed to report the type of animal that was mentioned in the news report as well as number of beeps. Importantly, children were instructed to focus most on the counting of beeps. The range of scores was between 0 and 20, as each trial included a score for the number of beeps as well as the type of animal.

### Basketball Task

Children were asked to shoot a basketball (440 g) from a standing position to a ring located 3.05 m away and 2 m high. Children were told that they would be given points depending on the outcome of each shot: 5 points were awarded for a successful shot that did not touch the backboard or ring (i.e., a “swish”), 4 points for a successful shot that touched the ring, 3 points for a successful shot that came off the backboard, 2 points for a miss that hit the ring, 1 point for a miss that hit the backboard and 0 points for any other miss. Children were provided with an opportunity at the beginning of day one to ask questions and clarify any aspects of the protocol that were unclear.

#### Procedure

The basketball shooting intervention consisted of a pre-test phase, a practice intervention, a post-test phase and a delayed retention test phase. Children were taken out of class individually during each phase to perform the task. The pre-test, practice intervention and post-test occurred on three consecutive days, whilst the retention test took place five-to-seven days after the post-test. The variation in days was a result of children being absent from school. Children were provided with five familiarization trials prior to the pre-test and the retention test.

**FIGURE 1:**
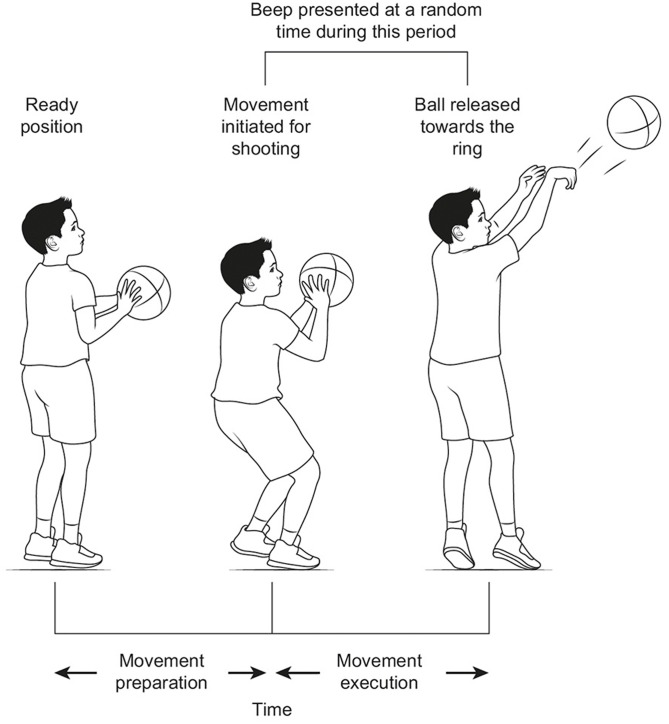
The sequence of events for the probe reaction time (PRT) conditions. Previous studies have differentiated between movement preparation and movement execution (Gray, 2004; Lam et al., 2010a,b). Our study specifically assessed PRT’s during the movement execution phase, which began when children initiated movement to shoot the ball (occurred after bouncing the ball). The beep was 80 ms in duration.

The three testing phases were comprised of the same conditions – a normal (single-task) condition, a probe reaction time (PRT) condition, and a dual-task condition. Each condition included 20 trials. The single-task condition required children to perform the task as per normal (i.e., no secondary task was provided). This was the primary measure of children’s learning. For the PRT condition, children performed the same basketball task, but were asked to say “yes” as quick as possible when they heard a loud beep. The auditory beep was 80-ms in duration and was presented via computer speakers on 12 randomly selected trials (**Figure 1**). The time of the beep was randomly dictated by the researcher (TB), but needed to occur after the child initiated movement for shooting (which typically involved the hands and ball lowering) and before the ball was released. Any beep that occurred earlier or later was removed from the analysis. Reaction times were recorded on a microphone (Phillips voice tracker) that was attached to the children’s shirt, and then measured using the computer program Audacity. For the dual-task condition, children performed the basketball task whilst simultaneously counting backward from 50. If children stopped counting, the subsequent shot was not recorded. Whilst discontinuing counting might reflect children’s WM being overloaded, it might also reflect attention being directed to the basketball task as opposed to the counting. We took the conservative option of only assessing basketball performance when children were counting, as we are confident that children’s WM was occupied when this occurred.

The practice intervention consisted of 12 blocks of 20 shots over three days. Day 1 involved the pre-test and 3 practice blocks, Day 2 involved 6 practice blocks, while Day 3 involved 3 practice blocks and the post-test. A 2 min break was provided between each practice block. Prior to every practice block, the researcher (TB) asked children to read five explicit instructions off an A4 sheet of paper (see **Table 2**). The instructions were designed to improve shooting mechanics and in turn shooting performance. The instructions were modified from a previous study with adults (Lam et al., 2009) and were developed in conjunction with an accredited junior basketball coach. After the instructions had been read aloud, the researcher asked children if the instructions made sense. If any did not, the researcher explained the instruction by asking questions such as: *“what do you think it means?”* and *“can you show me how you think you would do the instruction?”* This line of questioning continued until the child demonstrated an understanding for the instruction. Importantly, the researcher never provided a visual demonstration of the instruction and avoided explaining the instruction using other words.

**Table 2:** The five instructions that children read aloud prior to every practice block.

	Instructions
1	Bounce the ball on the ground twice before each shot
2	Start with your elbow under the ball
3	Use both hands to hold the ball but only shoot with one hand
4	Extend your arm fully when shooting
5	Finish the shot by pointing the shooting hand toward the rim

Practice blocks 2 and 11 also included PRT’s – the same protocol as the PRT condition during the testing phases. This provided an assessment of conscious processing during practice. All children performed the PRT task in blocks 2 and 11. Additionally, children were asked to recall the instructions at the beginning of days 2, 3, and 4 (retention) into a microphone.

#### Cover Story to Emphasize the Importance of the Instructions

The researcher (TB) devised a cover story and told the children that their points would be doubled if they shot with a good technique. Children were told that their technique would be compared to a professional basketball player via video replay, and if their technique was deemed similar they would receive double points. Indeed, a video camera was set-up on a tripod perpendicular to the child shooting the ball. Importantly, children were told that the instructions provided would help them shoot with a technique similar to a professional player. To reinforce this message, an A4 sheet of paper detailing the scoring system, as well the double points rule, was stuck on the basketball ring pole so that it was visible throughout the intervention. It must be emphasized, however, that no double points were included in the analysis of performance. This was merely a cover story designed to increase the likelihood that children would attempt to follow the instructions.

### Dependent Variables

There were five dependent variables:

#### Instruction Compliance

This was measured as the number of trials in which the child bounced the ball twice on the ground prior to shooting (as per instruction 1; see **Table 2**). The ‘bounce’ instruction was included as it allowed us to objectively measure whether the instruction was followed.

#### Recall of Instructions

This was defined as the number of instructions that children could recall at the beginning of each day. Instructions did not need to be recalled ‘word-for-word’; instead, children simply needed to state the main aspect of the instruction.

#### Shooting Technique

This was defined by a score, with points given for the execution of key technical points. The checklist of technical points was based on the four technical instructions (i.e., not including the “bounce” instruction). For every trial, children were given a 1 or a 0 for each technical point depending on whether their movements corresponded with the criteria; hence the maximum score for each trial was 4. A total technique score was computed for the pre-test, the post-test and the retention test. Importantly, technique was assessed by a person who was independent of the research aims. Technique for each child was then reanalysed by a second independent assessor for reliability purposes. Intra-class correlation coefficients indicated moderate-to-high correlations for total technique score (ICC = 0.85, *p* < 0.01).

#### Shooting Performance

This represented the number of points scored for each block of 20 shots. A score between 0 and 5 was recorded for every trial. Hence shooting performance strictly referred to performance outcome as opposed to movement mechanics.

#### Probe Reaction Time (PRT)

This was defined as the time duration (ms) between the onset of the beep and the onset of “yes” by the child. In situations where the child did not respond to the beep, the trial was removed from analysis. This occurred on 36 occasions (1.3% of total PRT trials) across 9 participants. Seven of these participants were in the lower WM capacity group. Of the 36 occasions where there was no response, 26 were from 2 participants – both of whom were in the lower WM capacity group.

### Statistical Analysis

Linear mixed modeling was used to estimate the association between group and each dependent variable: instructions recalled, compliance with instructions, shooting technique score, shooting performance and PRT’s. Each model included fixed effects for the intervention group, time period, and their interaction. Normally distributed random effects for subject were used to account for the within-subject correlation induced by the repeated measures experimental design. When the outcome was shooting technique score, shooting performance or PRT’s, normal residual error was used. For the count outcome of instructions recalled and instruction compliance, the model family was a Poisson with a log link. Likelihood ratio tests were used to test for the significance of the fixed effects (i.e., the interaction between group and time). The likelihood ratio test was performed with a Chi-square distribution using the appropriate degrees of freedom for the comparisons being made. Assessments about the magnitude of effects between groups were based on linear contrasts of the model fixed effects, and their 95% confidence intervals and *p* values using Holm’s method to adjust for multiple comparisons^1^. Cohen’s *d* effect sizes accompany *p* values for all pairwise comparisons. The assumptions of linearity and homoscedasticity for the mixed models were checked by inspecting residual plots, whilst the assumption of normality was assessed by observing histograms and qq-plots. All analyses were performed in the R (R Core Team, 2014) language using the *lme4* package (Bates et al., 2015) for the mixed modeling.

## Results

### Instruction Compliance

Both groups displayed compliance with the “bounce” instruction throughout the practice period. While the higher WM capacity group tended to complete the “bounce instruction” more than lower WM capacity group throughout the intervention, the difference between groups was not significant. Across the 12 blocks, the high WM capacity group completed this instruction on an estimated 56% of the trials (95% CI [33%, 95%]), whereas the low WM capacity group completed the instruction on an estimated 27% (95% CI [16%, 47%]) of the trials (*p* = 0.14, *d* = 0.64). Closer inspection revealed that the difference between the two groups became progressively less, with the estimated difference between the two groups being 26% (95% CI [-45%, 98%]) during Block 1 (*p* = 0.14, *d* = 0.75), compared to 20% (95% CI [-66%, 107%]) during Block 12 (*p* = 0.25, *d* = 0.45).

### Instructions Recalled

The higher WM capacity children consistently verbalized more instructions than the lower WM capacity children. On day two, the mean number of instructions recalled was 3.6 (95% CI [2.9, 4.4]) in the higher WM capacity group and 2.5 (95% CI [1.9, 3.2]) in the lower WM capacity group. The higher WM capacity group recalled a similar number of instructions on day three (3.9 instructions, 95% CI [3.2, 4.9]), whilst the low WM capacity group increased the number of instructions recalled (3.5 instructions, 95% CI [2.8, 4.3]). During the retention test, the high WM capacity group recalled most of the instructions (4.2 instructions, 95% CI [3.4, 5.1]), whereas the low WM capacity group only recalled 2.7 instructions (95% CI [2.1, 3.5]). The estimated difference between the groups was 1.1 instructions (95% CI [-0.1, 2.2]) on day 2 (*p* = 0.06, *d* = 1.3), 0.5 instructions (95% CI [-1.0, 1.9]) on day 3 (*p* = 0.41, *d* = 0.46), and 1.5 instructions (95% CI [0.1, 2.9]) during the retention test (*p* = 0.01, *d* = 1.38). However, care should be taken in concluding that the number of instructions recalled was influenced by a Group x Time interaction, as the removal of the interaction from the linear mixed model did not have a significant influence on the goodness of fit, as indicated by a likelihood ratio test [χ^2^(2) = 2.30, *p* = 0.31].

### Shooting Technique

The difference in technique score between the two groups was not significant during each testing phase, with the higher WM capacity group scoring an estimated 1 point less during the pre-test (95% CI [-16.4, 14.3], *p* = 0.91, *d* = 0.06), 5 points more during the post-test (95% CI [-21.3, 9.5], *p* = 0.91, *d* = 0.32), and 6 points more during the retention test (95% CI [-8.8, 21.9], *p* = 0.91, *d* = 0.41). Nonetheless, the higher WM capacity group significantly improved their technique score from pre-test to retention test by an estimated 12 points (95% CI [-3.5, 27.3], *p* < 0.001, *d* = 0.71), whereas the lower WM capacity group only improved their score by an estimated 5 points, which was not significant (95% CI [-3.7, 14.4], *p* = 0.54, *d* = 0.34). Essentially, both groups were executing, on average, 2 of the instructions during the pre-test, and this increased to almost 3 of the instructions during the retention test. However, removing the Group x Time interaction from the linear mixed model did not have a significant influence on the goodness of fit, as indicated by a likelihood ratio test [χ^2^(2) = 3.74, *p* = 0.15]; hence, care is warranted in concluding that technique score was influenced by a Group x Time interaction.

### Shooting Performance

**FIGURE 2:**
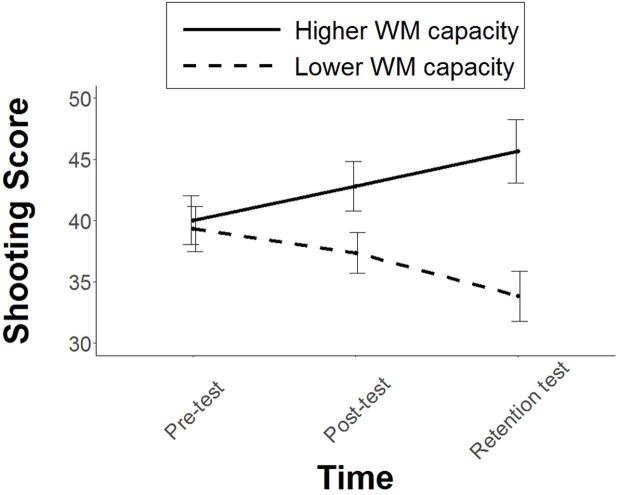
Mean shooting performance score for the two groups during the three stages of testing. Error bars represent standard error of the mean.

Our primary assessment of shooting performance only included the single task condition at each testing phase. Although minimal differences in shooting performance were apparent at pre-test (estimated difference = 0.7 points, 95% CI [-7.7, 9.1]) *p* = 0.81, *d* = 0.08), the higher WM capacity group tended to perform better than the lower WM capacity group during the post-test (estimated difference = 5.4 points, 95% CI [2.9, 13.8]) *p* = 0.06, *d* = 0.63) and this difference became more pronounced during the retention test (estimated difference = 11.8 points (95% CI [3.4, 20.2], *p* < 0.001, *d* = 1.04). The higher WM capacity group improved by 5.6 points from the pre-test to the retention test (95% CI [0.0, 11.3], *p* = 0.04, *d* = 0.45), whereas performance declined by 5.5 points for the low WM capacity group (95% CI [0.1, 11.1], *p* = 0.21, *d* = 0.59). A likelihood ratio test revealed that the interaction in our model (Group x Time) had a significant effect on shooting performance across the three testing phases [χ^2^(2) = 15.867, *p* < 0.001]. The group differences are illustrated in **Figure 2**.

We also predicted that the difference between higher and lower WM capacity groups would be evident immediately following the initial exposure to the instructions. This was tested by comparing shooting performance during the pre-test with performance during the first practice block. Counter to our hypothesis, however, the introduction of the instructions had no effect on shooting performance, with the higher WM capacity group scoring 1 point less in Block 1 compared to the pre-test (95% CI [-5.9, 3.7], *p* = 0.63, *d* = 0.13) and the lower WM capacity group scoring 3 points less in Block 1 compared to the pre-test (95% CI [-7.9, 1.6], *p* = 0.25, *d* = 0.38). Indeed, removal of the interaction (Group × Time) from the linear mixed model had no significant effect on the goodness of fit, as evidenced by a likelihood ratio test [χ^2^(1) = 0.66, *p* = 0.41].

### Dual-Task Performance

Neither group showed a significant decline in performance under dual-task conditions or PRT conditions (*p* > 0.05). The estimated difference between performance on the single-task and dual-tasks conditions across the three testing phases ranged between –1.3 and 4.6 points for the higher WM capacity group and between 0.8 and 3.8 points for the lower WM capacity group. Likewise, the estimated difference between performance on the single-task and PRT conditions ranged between 0.4 and 4.9 points for the higher WM capacity group and between 0.8 and 3.0 points for the lower WM capacity group.

### Probe Reaction Times (PRT)

**FIGURE 3:**
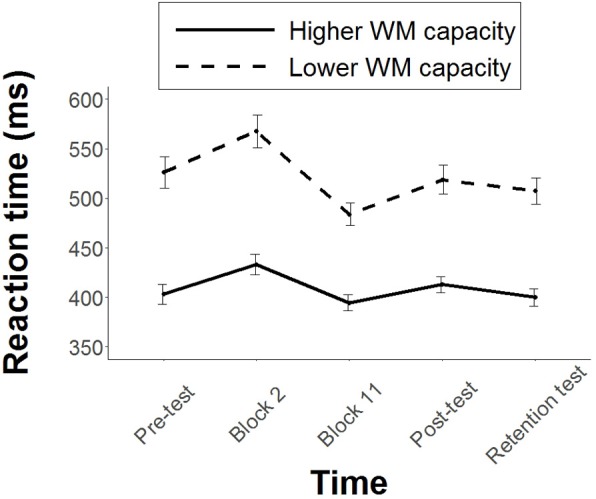
Mean PRT’s for each group throughout the study. Error bars represent standard error of the mean.

The lower WM capacity group displayed slower PRT’s than the higher WM capacity group throughout the study. The estimated difference between the groups was 127 ms (95% CI [1, 253]) at pre-test, 144 ms (95% CI [18, 270]) during Block 2, 91 ms (95% CI [34, 217]) during Block 11, 111 ms (95% CI [14, 237]) during the post-test and 122 ms (95% CI [3, 248]) during the retention test. Both groups recorded slower PRT’s in Block 2 compared to the pre-test, and faster PRT’s in Block 11 compared to Block 2. For the higher WM capacity group, PRT’s increased significantly from pre-test to Block 2 by 30 ms (95% CI [14, 75], *p* = 0.03, *d* = 0.40), and decreased significantly from Block 2 to Block 11 by 39 ms (95% CI [5, 84], *p* = .01, *d* = 1.15). For the lower WM capacity group, PRT’s increased significantly from pre-test to Block 2 by 47 ms (95% CI [2, 93], *p* = 0.003, *d* = 0.39), and decreased significantly from Block 2 to Block 11 by 92 ms (95% CI [47, 138], *p* = 0.0004, *d* = 0.55). Hence it appeared that both groups focused on the instructions more during early practice compared to late practice (see **Figure 3**). Given the similar PRT trends observed for both groups, it was no surprise that a likelihood ratio test showed that the removal of the interaction (Group × Time) from the linear mixed model had no significant effect on the goodness of fit [χ^2^(4) = 7.69, *p* = 0.10].

## Discussion

This study aimed to examine whether children with lower WM capacity were disadvantaged when learning a gross motor skill when practice placed high demands on WM. We hypothesized that heightening the demands on WM via the provision of five explicit technical instructions would lead to differences in basketball shooting performance between children of higher and lower WM capacity. The results supported our prediction, as children with higher WM capacity displayed continued improvement in shooting performance throughout the testing phases, whereas the opposite trend was apparent for children with lower WM capacity.

The contrasting performance profiles across the testing phases between the higher and lower WM capacity groups suggests that WM capacity influences motor learning when multiple explicit instructions are repeatedly delivered. We suspect that the higher WM capacity group was using the instructions to aid performance, as evidenced by their larger increase in technique scores from pre-test to the retention test. Indeed, this infers that the higher WM capacity group was more closely emulating the movement pattern as detailed by the instructions. Comparatively, the lower WM capacity group did not display a significant improvement in technique score. It seems likely that the children in the higher WM capacity group possessed greater ability to hold the instructions in an active state in mind whilst performing the 20 trials during each block. Hence, this afforded the opportunity to continually practice implementing the instructions. The lower WM capacity group, however, was probably less able to maintain attention on the instructions throughout each practice block. The interference caused by performing the basketball task likely impaired ability to maintain attention on the instructions. This explanation conforms to the attention control definition of WM capacity, in which larger capacity represents greater ability to control attention in the face of interference (Kane et al., 2001).

In understanding the results of our study, it is important to emphasize the effect of providing multiple instructions, as opposed to providing one instruction that directs attention internally. Instructions that direct attention internally tend to demand WM more than instructions that direct attention externally. An internal focus lends itself toward the conscious control of movements, which is cognitively demanding (Poolton et al., 2006b; Kal et al., 2013). However, a recent study of children revealed that verbal WM capacity was not predictive of performance improvements on a golf-putting task following either one internal instruction or one external instruction (Brocken et al., 2016). Hence, our findings appear to be the result of providing multiple internal instructions.

Certainly, our results are aligned with recent investigations exploring the relationship between WM capacity and the ability to enact instructions. It was demonstrated that when the volume of instructions was high (e.g., 6 items as opposed to 2 items), WM capacity correlated significantly with the ability to carry out the instructions (Jaroslawska et al., 2016; Waterman et al., 2017). Hence, WM capacity was positively associated with following instructions when the demands placed on WM were large. We extend this research by demonstrating that WM capacity is positively associated with the ability to carry out multiple instructions and consequently improve the outcome of a motor skill.

However, we are sceptical that this conclusion explains the result entirely as the difference in technique score does not explain why the lower WM capacity group displayed a negative learning trend. We suspect that the lower WM capacity children were attempting to follow the instructions in a step-by-step manner; however, due to their lower WM capacity (and hence reduced ability to control attention), the instructions were more likely to distract their attention away from important environmental cues. For instance, if looking at the target (i.e., the ring) is important for successful shooting (e.g., Vickers, 1996; de Oliveira et al., 2008; Wilson et al., 2009), then it is possible that children with lower WM capacity were less able to maintain focus on the target while simultaneously attempting to implement the instructions. Conversely, children in the higher WM capacity group were probably more capable of attending to multiple instructions whilst maintaining attention on important environmental cues. This implies that the process of updating movement patterns with multiple instructions is more difficult for individuals with a lower WM capacity.

Another explanation for the performance differences between the higher and lower WM capacity groups is the type of strategy adopted to use the instructions. It is possible that children in the higher WM capacity group selected more efficient strategies to deal with the instructions compared to children in the lower WM capacity group. Certainly, for cognitive tasks, such as arithmetic problem solving, individual differences in WM capacity have been related to strategy selection, which ultimately influences how efficiently problems are solved (Barrouillet and Lépine, 2005; Beilock and DeCaro, 2007). Moreover, the retrieval of information from long-term memory, such as the retrieval of the instructions during each practice block in the current study, requires WM and is influenced by strategy selection (Imbo and Vandierendonck, 2007; Unsworth, 2015). We therefore suspect one of two possibilities. Either children in the higher WM capacity group adopted more efficient strategies for using the instructions, or children in the lower WM capacity group adopted strategies that were too difficult to implement due to their lower WM capacity. For instance, attempting to implement multiple instructions during a single trial would be a more challenging strategy for children with lower WM capacity. This argument implies that optimal learning emerges when the performer adopts a strategy that reduces the likelihood of attention being diverted away from important environmental cues.

Our findings can also be explained by an embodied perspective of memory. Macken et al. (2015) proposed a limitless memory system that is the product of the dynamic interplay between a range of constraints, including material constraints (i.e., the information provided for the task), task constraints (i.e., the manner in which the task is to be completed), and repertoire constraints (i.e., the perceptual-motor and cognitive abilities of the individual). For instance, in the current study, the capacity to carry out the instructions was influenced by the type and volume of instructions that were provided (i.e., verbal instructions; material constraint), the requirements of what to do with the instructions (e.g., update movement patterns; task constraint), and the abilities of the performer (e.g., WM capacity, repertoire constraint). Accordingly, the combination of low WM capacity and a high volume of verbal instructions that necessitated updating movement patterns resulted in a poor ability to use the instructions, which ultimately impaired the learning experience.

An on-going issue with studies examining the effect of instructions on motor learning is identifying whether participants indeed follow the instructions (e.g., Buszard et al., 2013). Our data suggests that children in both groups were attempting to implement at least one of the instructions during practice. By way of example, children in both groups executed the “bounce” instruction throughout the practice intervention. Moreover, given that technique scores improved for both groups throughout the intervention, it appears that children from both groups were attempting to implement the instructions. Probe reaction times also increased after the initial presentation of the instructions (i.e., during Block 2) for both groups, suggesting that children were directing some attention toward the instructions during the early learning phase.

Contrary to our hypothesis, however, the dual-task results highlighted that most children did not become reliant on the instructions to shoot the basketball. During the post-test, only 20 of the 48 participants displayed poorer performance in the dual-task test. Likewise, only 22 participants scored fewer points under dual-task conditions in the retention test. Critically these participants were a mix of higher and lower WM capacity children. Thus, whilst children in the lower WM capacity group were presumably experiencing WM overload from instructions during practice, not all children became reliant on the instructions to perform the skill. Similarly, whilst the higher WM capacity group possessed greater ability to use the instructions effectively, only some children were seemingly reliant on the instructions in post-testing phases. This differs from research with adults, which consistently reveals the negative effects of explicit technical instructions on dual-task performance (e.g., Liao and Masters, 2001; Lam et al., 2009). Further research should explore whether age and/or cognitive development influences this occurrence.

We also hypothesized that differences in motor performance between higher and lower WM capacity groups would become apparent immediately after presentation of the instructions. This was based on the assertion that larger WM capacity would afford the ability to use the instructions immediately to augment performance. However, neither group displayed improved shooting performance during the first practice block. In fact, only 19 of the 48 children performed better during Block 1 compared to the pre-test, with 10 of these children coming from the lower WM capacity group and the remaining 9 children from the higher WM capacity group. We suspect that most children, irrespective of WM capacity, were overloaded during the first practice block, thereby resulting in no immediate performance gains.

The variation in the data also suggests that other factors, in addition to WM capacity, might have influenced shooting performance. For instance, the two groups differed in age, albeit only by half-a-year. This is likely a reflection of the relationship between age and cognitive development, with older children performing better on cognitive tasks (e.g., Gathercole et al., 2004; Luna et al., 2004; Luciana et al., 2005). It is important to note that our rationale for dividing children into lower and higher WM capacity groups based on measures of verbal WM capacity was due to previous findings that implicate the verbal system in working memory as the prominent construct influencing the ability to follow instructions (Jaroslawska et al., 2016). However, given that the two groups differed significantly in both verbal and visuo-spatial WM capacity, it seems that the major factor contributing to the motor learning differences in this study was the executive attention – the core function in measures of WM capacity (Kane et al., 2004). Nonetheless, the verbal component did appear to play a slightly more prominent role, as stronger correlations were revealed between learning (change in performance from pre-test to retention test) and verbal WM capacity (*r* = 0.51, *p* = < 0.001) than between learning and visuo-spatial WM capacity (*r* = 0.31, *p* = 0.03). Differences between the two groups were also observed for the two measures of attention (Score! and Score!DT). Interestingly, a stronger correlation was found between learning and the more complex measure of attention (Score!DT, *r* = 0.38, *p* = 0.006), as opposed to the simple measure of attention (Score!, *r* = 0.11, *p* = 0.46), therein providing further support for the executive attention argument. Score!DT required children to focus on counting beeps whilst simultaneously listening for a key word in a news report. Given the complexity of this task, which involves dividing attention whilst inhibiting distracting information from the news report, executive attention plays a critical role. Conversely, Score! simply involves sustaining attention on beeps with little involvement of executive attention. We therefore suspect that the executive attention component of working memory is the driving factor influencing motor learning when high loads are placed on working memory via explicit instructions.

Finally, we must not discount the possible influence of individual differences in processing speed. Processing speed refers to the time required to execute cognitive operations (Kail and Salthouse, 1994). Faster processing speed would therefore enhance the ability to implement multiple instructions whilst executing a motor skill. Whilst processing speed was not measured in this study, we did observe that the higher WM capacity group consistently displayed faster PRT’s than the lower WM capacity group (see **Figure 3**). This implies faster processing speed in the higher WM capacity group.

This study was not without its limitations, however. First, precise conclusions about the impact of instructions cannot be made without adequate control groups who receive no instructions. Certainly, the inclusion of such control groups would illuminate whether the instructions positively influenced performance. Second, no measures were in place to assess strategy use. Given that we suspect that children with higher WM capacity adopted more effective strategies when provided with multiple technical instructions, further research should test this hypothesis. Third, whilst the practice period was a similar length to many motor learning interventions, it was still relatively short in the context of acquiring a complex gross motor skill. Providing a longer practice period would offer insight into the effect of WM capacity on motor performance during both early and late learning. Currently we can only comment on the effect of WM capacity on early motor learning.

The practical implications from the research are clear. Placing an excessive burden on working memory resources will hinder learning by children with lower WM capacity. This may seem to be common sense, but the reality is that many practitioners (e.g., school teachers, rehabilitation specialists, sport coaches) rely on verbal instructions to teach new motor skills until competency is achieved. Future research should explore the effect of combining instructions with other teaching strategies, such as providing demonstrations (Obrusnikova and Rattigan, 2016), reducing errors (Capio et al., 2013a,b), or scaling equipment (Buszard et al., 2014). An interesting research question is whether a practical test can be developed for coaches to assess WM capacity. Current assessments of WM are unlikely to be adopted by coaches, but perhaps it is possible to estimate a person’s WM capacity by asking players to perform tasks in practice of varying instruction complexity.

## Conclusion

Assessing the influence of instructions on motor learning has a rich history, but surprisingly little research, if any, has investigated the mediating role of WM capacity. This line of research warrants further investigation given its practical relevance. Previous research has highlighted the strong relationship between WM capacity and the ability to implement instructions in a classroom setting (Engle et al., 1991; Gathercole et al., 2008; Jaroslawska et al., 2016), but this is the first study, to our knowledge, that has included a learning element. Much alike the studies that assessed the ability to carry-out instructions in a classroom, we found that the provision of multiple technical instructions, which seemingly placed high demands on WM, hindered motor learning for children with lower WM capacity. This supports the argument postulated by a number of researchers regarding the likely difficulties associated with explicit motor learning by individuals with comprised WM functioning (Steenbergen et al., 2010; Capio et al., 2012; Chauvel et al., 2012; van Abswoude et al., 2015). Critically, our assessment of additional variables, including attention and visuo-spatial WM capacity, suggests that executive attention ability, as opposed to specifically verbal WM capacity, is the driving factor influencing motor learning when high demands are placed on WM. Moving forward, we encourage researchers to account for individuals differences in cognitive variables, such as attention and WM capacity, when assessing motor skill acquisition in practice contexts that tax cognitive functions.

## Ethics Statement

This study was carried out in accordance with the recommendations of the National Statement on Ethical Conduct in Human Research (2007). All participants gave written informed assent and written informed consent was provided by their parents or guardians in accordance with the National Statement. The protocol was approved by the Victoria University Human Research Ethics Committee.

## Author Contributions

TB led the project, including designing the study, collecting and analyzing the data, and writing the manuscript. SV also administered data collection and contributed to writing the manuscript. DF, MR, JW, RP, FL, and RM contributed equally in designing the study and writing the manuscript. All authors approved the final version of the manuscript and have agreed to be accountable for all aspects of the work.

## Conflict of Interest Statement

The authors declare that the research was conducted in the absence of any commercial or financial relationships that could be construed as a potential conflict of interest.

## Footnotes

1 For instruction compliance, *p*-values were adjusted for three comparisons: difference between the two groups (i) across the entire practice period, (ii) during Block 1 and (iii) during Block 12. For instructions recalled, *p*-values were adjusted for three comparisons: difference between the two groups on day 2, day 4 and at retention testing. For shooting technique and shooting performance, *p* values were adjusted for nine comparisons: difference between the two groups during each testing phase and the difference between each testing phase within each group. For the assessment of dual-task performance, *p*-values were adjusted for four comparisons for the three testing phase: difference between the single-task and the dual-task conditions for each group and the difference between the single-task and the PRT condition for each group. For the separate analysis of shooting performance following immediate exposure to the instructions (i.e., comparing performance from pre-test to Block 1), *p*-values were adjusted for three comparisons: difference between pre-test and Block 1 for each group, and the difference between groups during Block 1. For the PRT data, *p*-values were adjusted for four comparisons: difference between pre-test phase and Block 2 for both groups and the difference between Block 2 and Block 11 for both groups.
